# Investigation of the Effects of Apigenin, a Possible Therapeutic Agent, on Cytotoxic and SWH Pathway in Colorectal Cancer (HT29) Cells

**DOI:** 10.34172/apb.2023.020

**Published:** 2021-11-28

**Authors:** Mustafa Cicek, Velid Unsal, Arif Emre, Adem Doganer

**Affiliations:** ^1^Department of Medical Biology, Faculty of Medicine, Kahramanmaras Sutcu Imam University, Kahramanmaraş, Turkey.; ^2^Department of Nutrition and Dietetics, Faculty of Health Science, Mardin Artuklu University, 47200, Mardin, Turkey.; ^3^Department of Surgery, Kahramanmaras Sutcu Imam University Faculty of Medicine, 46100, Kahramanmaras, Turkey.; ^4^Department of Biostatistics and Medical Informatics, Kahramanmaras Sutcu Imam University Faculty of Medicine, 46100, Kahramanmaras, Turkey.

**Keywords:** Apigenin, Colorectal cancer, The Salvador–Warts–Hippo (SWH) pathway, HT29 Cell line

## Abstract

***Purpose:*** Colorectal cancer (CRC) is one of the most common and fatal malignancies in humans, still leading to serious morbidity and mortality. We here aimed to investigate the effects of flavonoid apigenin, which is considered to have anti-tumoral activity on CRC with high epidemiological prevalence, on cell proliferation and cell survivals, and the positive and negative dose-dependent effects of genetic or mutational alterations in SWH pathway components on HT29 CRC cell lines.

***Methods:*** Human colon cancer cell lines HT-29 were commercially available. In each flask, 5 groups were formed, each of which consists of 5,000 cells for different dose groups and the cells were plated. After a 24 and 48 h incubation period, cytotoxicity values were measured by MTT assay and gene expression was assessed by real-time polymerase chain reaction (PCR) analysis method.

***Results:*** Application of 12.5 and 25 nM of apigenin significantly increased cell death in HT29 cell lines. LATS1, STK3 and TP53 gene expression decreased in the same dose groups compared to control and other groups.

***Conclusion:*** It has been concluded that TP53 gene is strongly correlated with LATS1 and STK3 genes among the SWH pathway factors in the progression of CRC and could be used as an important marker for early detection of malignant transmission. In addition, it may be effective in CRC cases especially when 25 nM of apigenin applies for therapeutic purpose.

## Introduction

 Colorectal cancer (CRC) is one of the most common and fatal malignancies in humans across the world. Approximately 1.2 million new cases of CRC occur each year. While roughly 90% of patients who undergo surgery at the early-stage ensure a long survival and a regular comfort of social life, the surgical interventions performed may be insufficient at the late stages when the prognosis is poor.^1^ Therefore, it is very important to investigate the genetic factors that are responsible for the changes in the expression of molecular markers of CRC in order to prevent cancer from developing. Natural compounds that have protective or disease-preventive properties are called phytochemicals. In this context, non-nutritive polyphenols found in fruits and vegetables are considered as the most studied phytochemicals.^2^ Of these phytochemicals, apigenin is known as one of the natural polyphenolic compounds in fruits and vegetables. Using different methods, it has been reported that apigenin exerts pharmacological effects pharmacologically as antioxidant, antiviral, antitumor and anti-inflammatory agents.^3,4^ Colorimetric viability assay, developed by Mosmann in 1983, is a common method used in the evaluation of anti-cancer efficacy. This assay is based upon the discoloration of the MTT molecule to which live cells are exposed.^5^ Nowadays the MTT assay is still one of the most widely used assays for assessing the activity of potential anticancer compounds and examining their interaction.^6^ In recent genetic studies, the Salvador–Warts–Hippo (SWH) signaling pathway has been described as a regulator of many cellular functions such as apoptosis, proliferation, organ and tissue growth.^7^ The activity of the SWH signaling pathway has undergone a substantial change in cancer cells in contrast to normal cells. When the SWH pathway is activated, it inhibits cell proliferation and promotes cell death and differentiation through a kinase cascade whose mechanism is well known.^8^ The major components of the SWH pathway consist of the transcriptional activators of genes that encode serine/threonine kinases (MST-1 and MST-2), large tumor suppressor 1 and 2 (LATS-1 and LATS-2), yes associated protein 1 (YAP-1), and Salvador homolog 1 (SAV-1).^9^ Although the mechanism of regulation of the SWH pathway is not fully known, the deregulation or genetic disruption in SWH pathway components has been reported in some types of cancer, including CRC.^10^ TP53 is a tumor suppressor gene that encodes the P53 protein and is inactivated by mutation or deletion in many types of human cancer.^11^ It has been postulated that genomic deletion of the tumor suppressor genes causes to inhibit the neighboring genes necessary for the survival or proliferation of cells and make the cell vulnerable.^12^ This study aimed to investigate whether apigenin flavonoid has proliferative, cytotoxic and anti-tumoral activity on CRC, which is an important health problem, as well as to investigate the positive or negative effects of genetic or mutational changes of SWH pathway elements in these cancer cells.

## Materials and Methods

 This study was carried out in Kahramanmaras Sutcu Imam University, Faculty of Medicine, Department of Medical Biology and Kahramanmaras Sutcu Imam University, Biomedical Practice and Research Center. Ethics committee approval was not considered to be necessary since cell lines and kits were commercially available.

###  Chemicals

 Apigenin, one of the natural polyphenolic compounds, was purchased from Santa Cruz (Santa Cruz Biotechnology, CA). Dimethyl sulfoxide (DMSO) and 3-[4,5-dimethylthiazol-2-yl]-2,5-diphenyltetrazolium bromide (MTT) were purchased from Sigma (St. Louis, MO, USA). The apigenin compound was dissolved in DMSO. The obtained solution was stored at -20°C. RPMI-1640 medium, fetal bovine serum (FBS) and Trypsin/ EDTA were purchased from Gibco (Pittsburgh, PA). The first-strand cDNA synthesis kit, RNA isolation kit and PCR primary probes (tp53, LATS1 and STK3) were purchased from Qiagen (Qiagen, Germany).

###  Cell culture and treatment protocol

 Human colon cancer cell lines HT-29 were commercially available from the American Type Culture Collection (ATCC, USA).Cell lines were cultured in RPMI 1640 medium supplemented with 10% heat-inactivated FBS (Gibco BRL, Grand Island, NY, USA), 100 g/mL penicillin and 100 µg/mL streptomycin using a 5% CO_2_ incubator set at 37°C.Cultured cells were treated with predetermined concentrations of apigenin (6.25 µM, 12.5 µM, 25 µM, and 50 µM) dissolved in DMSO at different time intervals (24 and 48 hours).

###  MTT assay

 MTT assay was used to measure cell proliferation. Cells (100 µL) were cultured in 96-well plates and treated with concentrations of 6.25 µM, 12.5 µM, 25 µM, and 50 µM at 24 and 48 hours. Following this, 10 µL of MTT reagent (5 mg/mL) was added to each well. Subsequently, 10 µL of MTT reagent (5 mg/mL) was added to each well. The reaction was terminated within 2 hours with the addition of 100 µL of solubilization solution. The dissolved MTT formazan products were measured at 590 nm absorbance at 24 and 48 hours. In order to measure cell death, cells were stained with 0.4% Trypan blue for 5 minutes at room temperature (20ºC). Then, they were examined under a microscope. The number of viable cells was determined with Trypan blue. Blue stained dead cells were counted and proportioned to the total cell count.

###  RNA extraction

 HT-29 cells were seeded in 6-well plates at a density of 5 × 10^5^. Culture medium was replaced with serum-free culture medium after 24 hours. Predetermined concentrations of apigenin (6.25 µM, 12.5 µM, 25 µM, and 50 µM) were added to the medium, and the cells were harvested after 6 hours. After the culture medium was removed, the cultures were washed with cold PBS. Cells not treated with apigenin were used as the negative control. After micro centrifugation at 800 g for 5 minutes at 4^°^C, total RNA was isolated using the Qiagen RNA Isolation Kit by a device in accordance with the manufacturer’s protocol (Qiagen, Germany).

###  Quantitative real time polymerase chain reaction (RT-qPCR) analysis

 Strand cDNA synthesis system RT-PCR kit (Qiagen, Germany) was used to perform cDNA synthesis. PCR primers for Tp53, LATS1, STK3 and the reference gene (Beta-actin) are shown in Table 1. PCR amplification was conducted in a Rotor Gene Q (Qiagen, Germany) using the following parameters: first denaturation at 94^°^C for 5 minutes followed by 35 cycles of reactions of denaturation at 94^°^C for 30 seconds, annealing at 58^°^C for 45 seconds, extension at 72^°^C for 45 seconds, and last extension at 72^°^C for 5 minutes. The relative measurement of mRNAs for Tp53, LATS1, and STK3 was made using the ∆∆CT method via the reference gene beta-actin. Means and standard deviations were calculated from two independent treatments.

**Table 1:** List of primers used for real-time PCR analysis

**Genes**	**Primers**	**Sequences**
TP53	Forward Reverse	5'-ATGGAGGAGCCGCAGTCAGAT-3' 5'-GCAGCGCCTCACAACCTCCGTC-3'
STK3	Forward Reverse	5'-GCAAAAAGACAACCTATCCTGG-3' 5'-GGGGGATCAATAAATAGGCTTC-3'
LATS1	Forward Reverse	5′-TCATCAGCAGCGTCTACATCG-3′ 5′-TCCAACCCGCATCATTTCAT-3′
β-actin	Forward Reverse	5'- GCAAGCAGGAGTATGACGAG -3' 5'- CAAATAAAGCCATGCCAATC-3'

###  Statistical analyses

 Shapiro-Wilk test was used to determine whether data show normal distribution. One-way analysis of variance (ANOVA) was employed to compare dose groups. Levene’s test was used to test for homogeneity of variances. Dunnett test, Tukey HSD test, and Tamhane T2 test were used for multiple comparisons (post hoc). T test was used for 24- and 48-hour group comparisons. Statistical parameters were expressed as mean ± SD. *P*< 0.05 was considered statistically significant. IC50 values were calculated. R 3.3.2 software and IBM SPSS version 22 (IBM SPSS for Windows version 22, IBM Corporation, Armonk, New York, United States) were used to analyze data. Graphs were drawn with the GraphPad Prism 9.2 program.

## Results

###  In vitro cytotoxicity results

####  Cell viability, proliferation and median inhibitor concentration results

**Figure 1:**
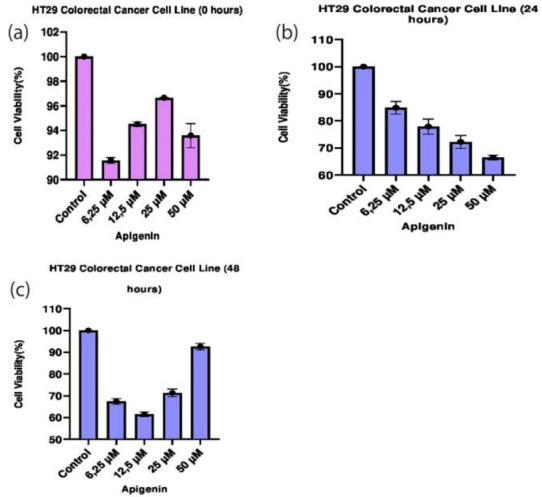
Effects of apigenin on the viability of HT29 cells. (a. 0. hours, b. 24. hours c. 48. hours). MTT shows significant differences between experimental groups compared to control.

 The dose-dependent cytotoxic effect of apigenin on CRC HT29 cell line cell proliferation was analyzed by MTT assay. Viability rates of cells treated with apigenin at concentrations of 6.25 µM, 12.5 µM, 25 µM, and 50 µM were assessed at the end of 0^th^, 24^th^ and 48^th^ hours (Table 2). Inhibitory effect of apigenin at these concentrations was compared with the control groups and between each other (Figure 1).

**Table 2:** Comparison of apigenin administration in HT29 cells both at different times and between different dose groups

**Hour**	**Apigenin HT29 Colorectal Cell Line**					***P***
	**Control**	**6.25 µM**	**12.5 µM**	**25 µM**	**50 µM**	
	**Mean±SD**	**Mean±SD**	**Mean±SD**	**Mean±SD**	**Mean±SD**	
0	100.00 ± 0.00^b,c,d^	91.56 ± 0.24^a,c,d^	94.51 ± 0.18^a,b,d^	96.66 ± 0.05^a,b,c^	93.59 ± 0.98	< 0.001*
24	100.00 ± 0.00^b,c,d,e^	84.85 ± 2.33^a,c,d,e^	77.88 ± 2.78^a,b,e^	72.24 ± 2.40^a,b^	66.47 ± 2.90^a,b,c^	< 0.001*
48	100.00 ± 0.00^b,c,d,e^	67.44 ± 1.29^a,c,d,e^	61.55 ± 1.08^a,b,d,e^	71.35 ± 1.72^a,b,c,e^	92.62 ± 1.51^a,b,c,d^	< 0.001*

*Difference is statistically significant; ^a^ Differences with the control group are significant; ^b^ Differences with the 6.25 µM group are significant; ^c^ Differences with the 12.5 µM group are significant; ^d^ Differences with the 25 µM group are significant; ^e^ Differences with the 50 µM group are significant.

 A significant difference was observed between the dose groups in terms of the effects of apigenin on HT29 cells (*P* < 0.05). The results of statistical analysis show that the highest cell death was observed at the concentration of 50 µM at the end of the 24^th^ hour, while the maximum cell death was detected at the concentration of 12.5 µM by the end of the 48^th^ hour (*P* < 0.05). When the 24^th^ and 48^th^-hour data were examined together, the most effective dose was 12.5 µM and statistically significant (*P* < 0.05). At the end of the 48^th^ hour, it was observed that a dose of 50 μM increased the viability of the cancer cells, bringing to a level almost equal to the control group. Effects of apigenin on HT29 cells at the median inhibitory concentrations were determined after 0th, 24^th^ and 48^th^ hours (Table 2). The measurements made in the 0th, 24^th^, and 48^th^-hour groups show that more cell death occurred in cancer cells treated with apigenin at concentrations of 12.5 µM and 25 µM than that at IC50 concentrations (*P* < 0.05) (Table 3).

**Table 3:** Demonstration of IC50 concentration levels in cell proliferation and gene expression of therapeutic apigenin compound performed at different concentrations to HT29 cell lines

**Hour**	**Level**	**Unit**
**IC50 concentration of HT29 cell line**		
0	0.0046	µM
24	200.4	µM
48	2.82	µM
**IC50 concentration of STK3 gene expression**		
24	41.42	µM
48	52.88	µM
**IC50 concentration of LATS1 gene expression**		
24	Could not calculate	µM
48	29.96	µM
**IC50 concentration of TP53 gene expression**		
24	20.78	µM
48	91.15	µM

###  Gene expression results

 The effect of different concentrations of apigenin on LATS1, STK3 and TP53 gene expression levels in HT29 cells was analyzed by RT-qPCR method between both 24^th^ and 48^th^ hours and between control and dose groups. Effects of pre-treatment with apigenin on LATS1, STK3 and TP53 gene expression levels between the control group and other groups, and IC50 concentration values between 24^th^ and 48^th^ hour are given in Table 3.

###  LATS1 gene expression results in HT29 cells

**Figure 2:**
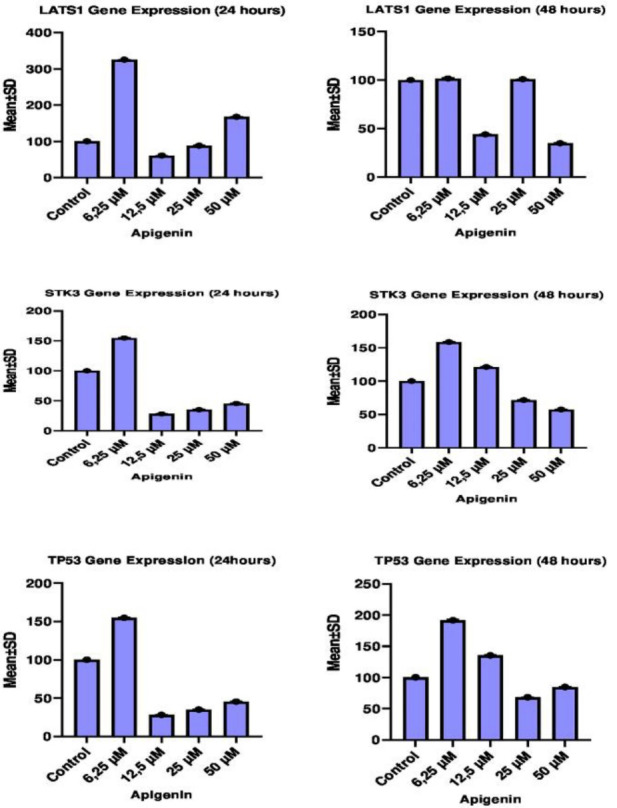
Comparison of 24 and 48 hours LATS1, STK3 and TP53 mRNA expression levels of all experimental groups with control.

 The 24^th^-hour treatment shows that LATS1 gene expression significantly increased in especially cells treated with 6.25 µM of apigenin compared to the control and other groups whereas LATS1 gene expression significantly decreased in cells treated with 12.5µM and 25 µM of apigenin in comparison to the control and other groups (*P* < 0.05).The 48^th^ hour treatment shows that LATS1 gene expression significantly increased in cells treated with 6.25 µM and 12,5 µM of apigenin compared to other groups (*P* < 0.05) but there was no significant difference in LATS1 gene expression between the same dose groups and control group (*P* > 0.05). LATS1 gene expression significantly reduced in cells treated with 25 µM and 50 µM of apigenin when compared with control and other groups (*P* < 0.05). When the 24^th^ and 48^th^-hour results were compared between each other, the most effective concentration of apigenin was 25 µM for the LATS1 gene (Table 4, Figure 2).

**Table 4:** Demonstration of the effect of apigenin on LATS1, STK3, TP53 gene expression between dose groups at 24 and 48 hours

	**Control**	**6.25 µM**	**12.5 µM**	**25 µM**	**50 µM**	***P***
	**Mean±SD**	**Mean±SD**	**Mean±SD**	**Mean±SD**	**Mean±SD**	
**Apigenin-treated HT29 colorectal cancer cell line LATS1 gene expression**						
24 h	100.00 ± 0.00^b,c,d,e^	325.47 ± 0.91^a,c,d,e^	60.87 ± 0.35^a,b,d,e^	88.03 ± 1.47^a,b,c,e^	167.67 ± 1.31^a,b,c,d^	< 0.001*
48 h	100.00 ± 0.00^c,d,e^	100.77 ± 0.31^d,e^	101.57 ± 0.70^a,d,e^	44.03 ± 0.51^a,b,c,e^	34.97 ± 0.71^a,b,c,d^	< 0.001*
**Apigenin-treated HT29 colorectal cancer cell line STK3 gene expression**						
24 h	100.00 ± 0.00^b,c,d,e^	114.87 ± 0.80^a,c,d,e^	28.57 ± 1.14^a,b,d,e^	34.23 ± 0.42^a,b,c,e^	73.60 ± 0.56^a,b,c,d^	< 0.001*
48 h	100.00 ± 0.00^b,c,d,e^	158.53 ± 0.78^a,c,d,e^	121.30 ± 0.40^a,b,d,e^	71.50 ± 0.82^a,b,c,e^	57.47 ± 0.93^a,b,c,d^	< 0.001*
**Apigenin-treated HT29 colorectal cancer cell line TP53 gene expression**						
24 h	100.00 ± 0.00^b,c,d,e^	154.80 ± 0.75^a,c,d,e^	28.07 ± 0.47^a,b,d,e^	35.07 ± 0.85^a,b,c,e^	45.37 ± 0.97^a,b,c,d^	< 0.001*
48 h	100.00 ± 0.00^b,c,d,e^	191.63 ± 1.89^a,c,d,e^	135.33 ± 1.21^a,b,d,e^	68.27 ± 0.47^a,b,c,e^	84.63 ± 1.20^a,b,c,d^	< 0.001*

*Difference is statistically significant; ^a^ Differences with the control group are significant; ^b^ Differences with the 6.25 µM group are significant; Differences with the 12.5 µM group are significant; ^d^ Differences with the 25 µM group are significant; ^e^ Differences with the 50 µM group are significant.

###  STK3 gene expression results in HT29 cells

 The 24^th^-hour treatment shows that STK3 gene expression significantly enhanced in especially cells treated with 6.25 µM of apigenin compared to the control and other groups whereas STK3 gene expression significantly reduced in cells treated with 12.5 µM and 25 µM of apigenin in comparison to the control and other groups (*P* < 0.05). The 48^th^-hour treatment indicates that SKT3 gene expression significantly increased in cells treated with 6.25 µM and 12,5 µM of apigenin compared to other groups (*P* < 0.05) while SKT3 gene expression significantly decreased in cells treated with 25 µM and 50 µM of apigenin when compared with the other groups and control group (*P* < 0.05). When the 24^th^ and 48^th^-hour results were compared between each other, the most effective concentration of apigenin was 25 µM for the STK3 gene (Table 4, Figure 2).

###  TP53 gene expression results in HT29 cells

 The 24^th^-hour treatment shows that TP53 gene expression significantly increased in especially cells treated with 6.25 µM of apigenin compared to the control and other groups but TP53 gene expression significantly reduced in cells treated with 12.5 µM, 25 µM and 50 µM of apigenin in comparison to the control and other groups (*P* < 0.05). The 48^th^-hour treatment reveals that TP53 gene expression significantly increased in cells treated with 6.25 µM and 12,5 µM of apigenin compared to other groups (*P* < 0.05) whereas TP53 gene expression significantly reduced in cells treated with 25 µM and 50 µM of apigenin compared to the other groups and control group (*P* < 0.05). When the 24^th^ and 48^th^-hour results were compared between each other, the most effective concentration of apigenin was 25 µM for the TP53 gene (Table 4, Figure 2).

## Discussion

 Stem cells present in the human body can be turned into different types of cells.^13^ Embryo-derived stem cells are classified as developmental or postnatal (organ or tissue-specific and adult stem cells) stem cells.^14^ Besides, the properties of the organ or tissue-specific stem cells affect the behavior of stem cells.^15^ Therefore, we preferred to use a commercially available HT29 CRC cell line so that our study is of good quality and our experimental results are reliable. Plant secondary metabolites that show different biological activities and have unique biological effects are called flavonoids.^16^ Recent chemo-preventive approaches have aimed to optimize and develop phytochemical therapeutic agents from plant-derived bioactive compounds that exert different therapeutic and pharmaceutical effects.^17^ Flavonoids are phenolics found in almost all fruits and vegetables and have been extensively investigated for their anticancer properties, including anti-invasive and anti-metastatic activities.^18^ Recent studies on CRC show that apigenin is a flavonoid compound that has the potential to prevent invasion and metastasis.^19^ Our study sought to reveal changes in cell proliferation, viability, and cytotoxicity as well as SWH pathway and tumor suppressor proteins when CRC cells are treated with apigenin under in vitro conditions. This study covering a wide range of concentrations reveals that cell viability declined significantly in HT29 cell lines in response to the high concentration of apigenin. IC50 values for all the different concentrations of apigenin in this cell line were calculated, and the selected range of apigenin concentration consisted of those above and below the IC50 molar concentrations specific to the HT29 cell line. In vitro cytotoxicity tests should be performed with cells homologous to human tissue.^20^ In addition, these tests should be simple, reproducible, affordable and appropriate to evaluate the basic biological properties of the tissue where the application will perform.^21^ A study reported that the viability of CRC cells could be effectively inhibited by the application of apigenin, resulting in chromosomal condensation and formation of apoptotic bodies.^22^ Another study found that the therapeutic application of AP7Glu reduced the viability of HCT116 CRC cells and increased cell death stimulation.^23^ Our study demonstrates that the therapeutic application of apigenin reduced the cell viability while increasing the cell death in HT29 cells and produced a cytotoxic effect in CRC cells under in vitro conditions. At time intervals of 24 and 48 hours, administration of high-dose apigenin caused more effective acute toxicity than the administration of low-dose apigenin. LATS kinases are a family of protein consisting of two isoforms, LATS1 and LATS2.^24^ LATS1 is a major component of the MST2 (STK3) pathway involved in apoptosis or cell cycle regulation, but it can regulate biological functions such as cell proliferation or cell migration, independently of this pathway.^25^ It was found that the LATS1 gene is important in the regulation of cancer development in mammalian cells and LATS1 expression is reduced in cancers.^26^ LATS SWH was defined as a central mediator in the tumor-suppressor pathway.^27^ Destruction of SWH pathway activity is considered to be implicated in the formation of multiple human cancers. Serine/threonine kinase-3 (STK3) and LATS1 gene are important factors related to signal transduction in the SWH pathway.^28^ Adenovirus-mediated overexpression of LATS1 gene in HeLa and LATS1-/- cells causes suppression of tumor formation through induction of apoptosis.^29^ LATS1 is considered to exert a suppressive role in some tumors. expression of LATS1 has been found to decrease in sarcomas, astrocytomas and soft tissue origin tumors.^26^ Recent studies have reported that LATS1 expression decreased in gastrointestinal system cancers but it was overexpressed in CRC.^8^ Our results indicate that LATS1 gene expression reduced in HT29 CRC cell lines in the administration of 12.5 nM of apigenin after 24 hours and in the administration of 25 and 50 nM of apigenin 48 hours after. LATS1 expression increased extremely in the administration of 6.25 of nM apigenin after both 24 and 48 hours. Reduced expression of the LATS1 gene, an important transformer of the SWH pathway, suggests being linked with the deregulation of the SWH pathway. Our findings are consistent with the decreased expression of LATS1 gene in cancerous tissues in many recent clinical studies. Therefore, we consider that the administration of therapeutic apigenin reduces LATS1 gene expression in cancer cells and plays a suppressive role in tumor formation. With the human genome sequence completed, it has been shown that 20% of genes encode proteins involved in signal transduction. More than 500 protein kinases among these genes were found to exert specific and reversible control over protein phosphorylation. Mutations or other genetic alterations result in dysregulation of kinase signaling and irregular expression or activity and are also associated with malignant transformation.^30^ STK3, also known as MST2, is a proapoptotic cytoplasmic kinase.STK3 is one of the main components of the mammalian SWH pathway that controls cell growth, proliferation, apoptosis and some stress responses.^31^ Multiple apoptotic agents induce STK3 activity in cultured mammalian cells and stimulate apoptosis through increasing the interaction of LATS1 and STK3.^32^ Serine/threonine kinases play an important role in transmitting phosphorylation signals of cell cycle regulators and many cytoplasmic and nuclear effector sequences through cellular homeostasis. Kinases are a very important target for therapeutic intervention in cancer because there is comprehensive information about how small molecules can be inhibited. Recently, some kinase inhibitors have been developed and some of them have been used in the medical field of application.^33^ Our ability to interfere with kinases is more than our knowledge of which kinase or kinases’ mechanisms are linked with which cancer type. A high-efficiency strategy has been adopted in recent studies in order to activate mutations or to activate kinases in the initiation and progression of cancer by identifying misregulated expression in gene profiling experiments.^34^ Activated MST2 p53 protein is stabilized by the LATS-1 gene and downregulation of MST2 gene expression is often seen in human CRCs.^29^ Our findings found that the expression of STK3 gene in HT29 CRC cell lines decreased significantly in applications of 12.5 nM and 25 nM of apigenin after 24 hours and in applications of 25 nM and 50 nM of apigenin after 48 hours. We believe that the STK3 and LATS1 genes among the SWH pathway factors show tumor suppressor properties in colon cancer cells since the decrease in their levels of gene expression is inter correlated. It is most likely that our natural compound, apigenin, inhibits the degradation of the SWH pathway through the reduction in STK3 (MST2) and LATS1 gene expression. The TP53 is a gene that most commonly mutates in human cancers and is exposed to loss-of-function in CRCs. The wild type TP53 gene produces DNA binding p53 protein, which acts as a transcriptional activator of genes that prevent the development.^35^ Under physiological conditions, cells stop their growth through induction or activation of p53 in response to DNA-damaging agents and other factors. Activated p53 induces various growth-limiting responses, including cell cycle arrest, apoptosis and differentiation in order to facilitate DNA repair.^36^ Many studies reported that patients with TP53 mutations have shorter survival rates compared to patients without mutations. The p53 gene is considered to be a highly selective and effective target for therapeutic intervention. Some of the new therapeutically developed therapies target TP53 mutant cells whereas others aim to directly correct TP53 mutations or to preserve and repair the integrity of the p53 pathway.^37^ Our findings revealed that there was a significant decrease in TP53 gene expression after 24 hours therapeutic administration of 12.5 nM, 25 nM and 50 nM of apigenin, and after 48 hours administration of 25 nM and 50 nM of apigenin. Consistent with the literature, our observation shows that there was an increase in the TP53 gene expression, proliferation and proliferation-related signaling kinases in CRC cells.TP53 produces these responses largely by altering the expression of the LATS1 and STK3 gene sequences known to be under the transcriptional control of the TP53 gene and reflecting mutational inactivation.

## Conclusion

 It has been concluded that the TP53 gene is strongly correlated with LAST1 and STK3 genes among the SWH pathway factors in the progression of CRC and could be used as an important marker for early detection of malignant transmission. In addition, the certain dose of apigenin may also be effective in the treatment of the CRC cases.

## Author Contributions

**Conceptualization: **Mustafa Cicek, Velid Unsal, Arif Emre.

**Data curation: **Mustafa Cicek, Velid Unsal, Adem Doganer.

**Formal Analysis: **Mustafa Cicek, Velid Unsal.

**Funding acquisition: **Mustafa Cicek, Velid Unsal, Adem Doganer, Arif Emre.

**Investigation: **Mustafa Cicek, Velid Unsal, Arif Emre.

**Methodology: **Mustafa Cicek, Velid Unsal, Adem Doganer.

**Project administration: **Mustafa Cicek.

**Resources: **Mustafa Cicek, Velid Unsal, Arif Emre.

**Software: ** Mustafa Cicek, Velid Unsal, Adem Doganer.

**Supervision: **Mustafa Cicek, Velid Unsal, Adem Doganer, Arif Emre.

**Validation: **Mustafa Cicek, Velid Unsal,Adem Doganer.

**Visualization: **Mustafa Cicek, Velid Unsal.

**Writing – original draft: **Mustafa Cicek, Velid Unsal, Adem Doganer, Arif Emre.

**Writing – review & editing: **Mustafa Cicek, Velid Unsal.

## Ethical Issues

 Not applicable.

## Conflict of İnterest

 The authors declare that they have no conflict of interest.
